# The effect of mandala coloring and free coloring on the happiness in veterans with post-traumatic stress disorder in the Covid-19 pandemic: a randomized clinical trial

**DOI:** 10.1186/s12888-024-05886-x

**Published:** 2024-06-25

**Authors:** Mohammad-Amin Nasiri, Seyedeh Azam Sajadi, Zahra Farsi, Marzie Heidarieh

**Affiliations:** 1 Medical Surgical Nursing Department, Nursing School, Aja University of Medical Sciences, Tehran, Iran; 2 Nursing Management Department, Nursing School, Aja University of Medical Sciences, Tehran, Iran; 3 Medical-Surgical Nursing, Research and Ph.D. Nursing Departments, Nursing School, Aja University of Medical Sciences, Tehran, Iran; 4 MSc, Clinical Psychology, Psychology Department, Milad Hospital, Tehran, Iran

**Keywords:** Happiness, Veterans, Art, Painting, Mandala, Stress disorder, Post-traumatic

## Abstract

**Background:**

Post-traumatic stress disorder (PTSD) can lead to complications such as depression and grief, which are more prevalent in veterans than in the general population. Recently, art-making, including mandala coloring, has gained attention as a potential treatment for PTSD patients.

**Methods:**

This randomized clinical trial was conducted on 84 male veterans diagnosed with PTSD and hospitalized at the Milad Psychiatric Center in Tehran, Iran. The patients were recruited using a convenience sampling method and were assigned to either the mandala coloring group or the free coloring group. The Post-Traumatic Stress Disorder Checklist DSM-5 and the Oxford Happiness Scale were used to collect data. The intervention group colored mandala designs, while the control group colored squares freely. Coloring was done twice a week for three weeks.

**Results:**

The mean baseline happiness scores did not differ significantly between mandala coloring group and free coloring group (*p* = 0.376). However, at the end of study, happiness scores were significantly higher in mandala coloring group than in free coloring group (*p* < 0.001). After the intervention, happiness score of both groups increased significantly (*p* < 0.001).

**Conclusion:**

Both coloring methods increased veterans’ happiness scores; however, mandala coloring was more effective than free coloring. It is recommended that art-making be added to conventional treatments for veterans with PTSD.

**Trial registration:**

This study was registered in Iranian Registry of clinical trials (No. IRCT20210604051491N1, 29/08/2021).

## Introduction

Post-traumatic stress disorder (PTSD) is a mental disorder that affects individuals who have been exposed to traumatic events such as natural disasters, serious accidents, rape, and war [1]. Studies have shown that there is a broad scope of chronic and acute complications such as PTSD in veterans [2, 3]. So that approximately 30% of individuals who have experienced traumatic incidents are likely to develop PTSD, with war veterans having an even higher risk [1].

The incidence of PTSD in chemical attack veterans of the Iran-Iraq war is reported to be 40%, and despite the war ending 35 years ago, this disorder remains one of the most common reasons for hospitalization among war veterans [4]. In a study, the prevalence of PTSD in the Iran-Iraq war veterans and prisoners was reported to be 51% and 79%, respectively [5]. This disorder causes intrusive thoughts, negative feelings, reluctance to remember the traumatic event, paroxysmal arousal and reactivity as well as depression, anxiety, isolation, difficulty concentrating, sleep problems, and reduced quality of life [6].

Happiness is a crucial aspect of human life, characterized by positive emotions, satisfaction with life, and the absence of negative emotions. It promotes energy, enthusiasm, vitality, activity, hope, and accelerates the healing process, protecting people against stress and ensuring their physical and mental well-being [7]. Based on the review studies, increasing happiness has been associated with increased mindfulness, reduced post-traumatic stress disorder symptoms, and overall improved quality of life. Therefore, identifying methods that promote happiness in patients is crucial [8, 9]. However, according to the World Happiness Report, after the outbreak of the COVID-19 pandemic and implementing strict health laws related to social distancing, mental health problems in the general population of countries have increased and their happiness levels decreased [10]. This situation was probably worse for veterans. Nofarsti et al. investigated the happiness level and factors affecting it in Iranian veterans and reported that happiness level in veterans was lower than the average of the general population [7]. In a study of Iranian athlete veterans, their happiness score was 58 out of 87, which was considered moderate; researchers also found a significant relationship between happiness and mental strength [11]. A study of American guard personnel also found an inverse association between the severity of PTSD symptoms and their happiness level [12].

For individuals experiencing mild PTSD symptoms, a watchful waiting approach with appropriate follow-up may be sufficient. However, for those with more severe or prolonged symptoms lasting over 4 weeks, psychological treatments like cognitive therapy and exposure-based therapy should be considered as the primary treatment option [13]. In cases of severe PTSD or for individuals who are unwilling to undergo psychological interventions, the use of antidepressants such as Selective serotonin reuptake inhibitors (SSRIs) is recommended [14]. However, more than a third of people with PTSD do not recover despite these treatments. Factors such as the severity, recurrence, and nature of the events in the war scene, and the complexity of such events, complicate treatment for veterans [15, 16]. Many veterans are unable to establish a good relationship with their therapist —which is necessary for cognitive behavioral therapies to be effective — hence, do not adhere to treatment properly [17]. Nowadays, complementary medicine approaches, particularly mind-body interventions, are being considered as alternative treatments for patients with mild forms of PTSD, as well as an adjunct to recovery and improvement for patients with moderate to severe PTSD [18].

Art therapy, as defined by the British Association of Art Therapists, is a form of psychotherapy that utilizes artistic media as its primary mode of communication. The ultimate goal of art therapy is to facilitate personal growth and change in patients through the use of artistic materials [19]. Art therapy utilizes various artistic modalities such as music, poetry, painting, drawing, sculpturing, etc [20]. Several studies indicate the effectiveness of art-making methods [21–23] and positive interventions [7, 24, 25] in reducing symptoms of patients with mental disorders (such as anxiety, depression) and improving mental health and well-being, and have reported these methods facilitate the expression and control of emotions, improve happiness and life satisfaction in various people, including soldiers and veterans [26–28]. While art-making has benefits, there are concerns about its potential negative impacts, such as stress and exacerbation of disease symptoms from recalling past memories and it being a distraction rather than therapeutic. So, it is advised that art-making be supervised by a mental health therapist [29, 30].

Coloring mandala patterns is one of the common art-making methods. Mandala patterns include circular and geometric designs and are frequently used as a tool for meditation, to promote psychological healing, and integration of mind and body [28].

The roots and origins of mandala patterns are in Hindu and Buddhist religions, serving as symbols and representations of the world and as tools for reflection and inner exploration in humans [31]. Although the beginnings and use of mandala patterns were initially more concentrated in East Asian countries such as India, China, and Japan, the use of these patterns in various cultures and countries is clearly observable. For example, designs used in architecture and cultural spaces in countries like Iran and Turkey, as well as churches in Europe, all have mandala patterns [32, 33]. Therefore, it seems that the popularity of mandalas continues globally, and individuals with diverse backgrounds appreciate their beauty, symmetry, and spiritual significance.

By drawing and coloring these patterns, people can become aware of and communicate with their feelings and thoughts and focus their attention on the present moment. Mindfulness and emotional self-regulation are then improved, and anxiety reduced [34].

A study of graduate students in counseling showed that coloring a mandala for 12 min at the beginning of class reduced anxiety more effectively than free coloring [34]. In another study, Alloway et al. investigated the effect of two 15 min sessions of simple coloring and mandalas on stress and anxiety levels in eight veterans with PTSD and reported that coloring significantly reduced anxiety and stress scores in veterans regardless of type or conditions. Despite these findings, some researchers reported that such short-term interventions are unlikely to have lasting effects and that additional studies are needed [35–37]. However, no studies have examined the effects of coloring, particularly mandala coloring, on veterans’ happiness.

War-related trauma such as PTSD can lead to complications such as anger, depression, painful memories, sleep disturbances, suicidal thoughts, and grief. These complications disrupt the patient’s relationship with their family and others, and reduce the individual’s quality of life. Reports also show that veterans are less happy than the general population. Also, given the prevalence of PTSD in veterans and the limited evidence on the effectiveness of art-making methods in veterans suffering from PTSD, and at the peak of the fourth wave of COVID-19, this study was conducted with the aim of comparing free and Mandela coloring on the level of happiness among veterans suffering from PTSD.

## Methods

### Design and setting

A randomized, clinical trial was conducted from 01-09-2021 to 15-04-2022. It was registered in Iranian Registry of clinical trials (No. IRCT20210604051491N1, 29/08/2021).

The study was conducted on veterans diagnosed with PTSD and hospitalized at Milad Psychiatric Center in Tehran, Iran. This medical center has two wards for veterans with PTSD. The two wards are similar in terms of physicians, health care providers, physical space, and types of patients. Therefore, one of the wards was randomly assigned to mandala coloring and the other to free coloring. For this purpose, wards numbers written on two separate balls and placed inside a bag. The first ball selected would determine the control group, while the second ball would signify the intervention group. Randomization was done by the first author.

### Sampling

The results of a previous study examining the effect of mandala art-making on anxiety in women with substance abuse were used to calculate the sample size. The mean posttest anxiety of the intervention and control groups was reported to be 1.69 ± 0.29 and 1.93 ± 0.36, respectively [38]. Using the formula for comparison between two means, and considering a type I error of 0.05, a type II error of 0.1, μ1 of 1.69, μ2 of 1.93, ${s}_{1}$ of 0.29, and ${s}_{2}$ of 0.36, the sample size was calculated to be 39 per group. However, given a possible dropout of 10%, we recruited 42 patients in each group by convenience sampling method.

$$n= \frac{{\left({z}_{1-\frac{\alpha }{2}}+{z}_{1-\beta }\right)}^{2}{({s}_{1}+{s}_{2})}^{2}}{({{\mu }_{1}-{\mu }_{2})}^{2}}=\frac{{(1.96+1.28)}^{2}{(0.29}^{2}+{0.36}^{2})}{{(1.69-1.93)}^{2}}=42$$

Inclusion criteria were a medical diagnosis of PTSD and obtaining a minimum score of 50 from the Post-Traumatic Stress Disorder Checklist for DSM-5 (PCL-5), no physical disorders (including visual impairment such as color blindness, presbyopia, etc. and no finger or hand movement disorders affecting coloring), no psychotic disorders (schizophrenia, no suicidal or homicidal thoughts, no crisis in the past six months, including the death of relatives or divorce), age < 65 years, and no self-reported substance abuse. Inclusion criteria were checked based on the patient self-reports and medical records. The exclusion criterion was the patient’s decision to withdraw from the study and aggravation of the patient’s condition during the intervention based on the psychologist’s opinion and occurring visual disturbances or problems in hand movements during the intervention.

The intervention group participated in mandala coloring and the control group participated in free coloring.

### Data collection instruments

The study data was collected using three questionnaires: the individual characteristics questionnaire, the PCL-5, and the Oxford Happiness Scale (OHS). The individual characteristics questionnaire included questions on the patient’s age, education level, duration of suffering from PTSD, marital status, number of children, physical disability.

The PCL-5, developed by Weathers, was used to assess PTSD symptoms. It consists of 20 self-reported items, scored on a 5-point Likert scale ranging from “zero: Not at all” to “four: Extremely.” The sum of the items produces a symptom severity score between zero and 80, with a score of 50 indicating acute PTSD [39]. The first researcher was present with the veterans when completing the PCL-5 to aid them in understanding items and answer their queries, if any. An earlier study in Iran confirmed the validity and reliability of the PCL-5 and reported its Cronbach’s alpha as 0.79 [39]. In the current study, the Cronbach’s alpha for PCL-5 was 0.8.

The OHS was developed by Argil et al., [40]. The scale includes 29 items scored on a four-point Likert scale. The OHS minimum and maximum scores are zero and 87, respectively. Each item consists of four sentences, with the first sentence scoring zero and the fourth sentence scoring three. Scores < 22, 23–44, 45–66, and 67–87 are considered as low, moderate, high and very high happiness, respectively [41]. Argil et al. reported the Cronbach’s alpha of this scale as 0.90 [40]. In Iran, Alipour et al. examined the content validity and reliability of the OHS and reported its Cronbach’s alpha as 0.93 [42]. In the current study, the Cronbach’s alpha for OHS was 0.84.

### Intervention

At Milad Psychiatric Center, veterans attend group therapy sessions twice a week for one-and-a-half hours each. During these sessions, a psychologist teaches them stress management techniques such as body scanning, slow breathing, and muscle relaxation. These group therapy sessions are considered routine care. After reviewing previous studies [35, 43–45], the intervention was designed as follows. Moreover, previous studies had limited mandala coloring sessions to one or two, whereas in the current study, the number of sessions was increased to six. This adjustment aimed to explore the long-term effects of mandala coloring on a population of veterans. The first author and the psychologist of the study were responsible for the implementation of the interventions.

The intervention and data collection took place over five consecutive weeks. In the first week, patients provided informed consent and underwent a pre-test. Mandala coloring was done in the intervention group during the second to fourth weeks, while the control group engaged in free coloring. The post-test was conducted in the fifth week.

Before the intervention, participants in the mandala coloring group received a training session on mandala designs and coloring techniques. At the end of each group therapy session, every veteran received 12 soft crayons and an uncolored, structured 20 × 20 cm mandala design to color within 15 min. This process was repeated for three consecutive weeks. In the control group, veterans were provided with 12 crayons and an A_4_ paper with a blank 20 × 20 cm square to color freely for 15 min during each session. The coloring of mandala patterns was done six times by veterans during three weeks.

Coloring sessions were held asynchronously for the two groups to reduce the risk of data contamination between the two groups. The participants completed the individual characteristics questionnaire and PCL-5 one week before the study, but they completed the OHS both one week before and one week after the last coloring session. to evaluate the long-term effects of coloring, post-test was performed one week after the last coloring session in each group. None of the participants dropped out during the study.

### Ethical considerations

This study was approved by the supreme Research Council and the Ethics Committee of the Aja University of Medical Sciences (No: IR.AJAUMS.REC.1400.054). Permission to conduct the study was obtained from the officials of the Aja University of Medical Sciences and presented to hospital and departmental officials where the study was conducted. The objectives of the study were explained to all participants, and they signed the informed consent form. Participants were assured that they could withdraw from the study at any time, participation in this study was voluntary and would not harm them. They were also assured that their information would be kept confidential and that the results of the study would be provided to them upon request. The rights of all participants were respected in accordance with the Helsinki Declaration.

### Data analysis

**Fig. 1:**
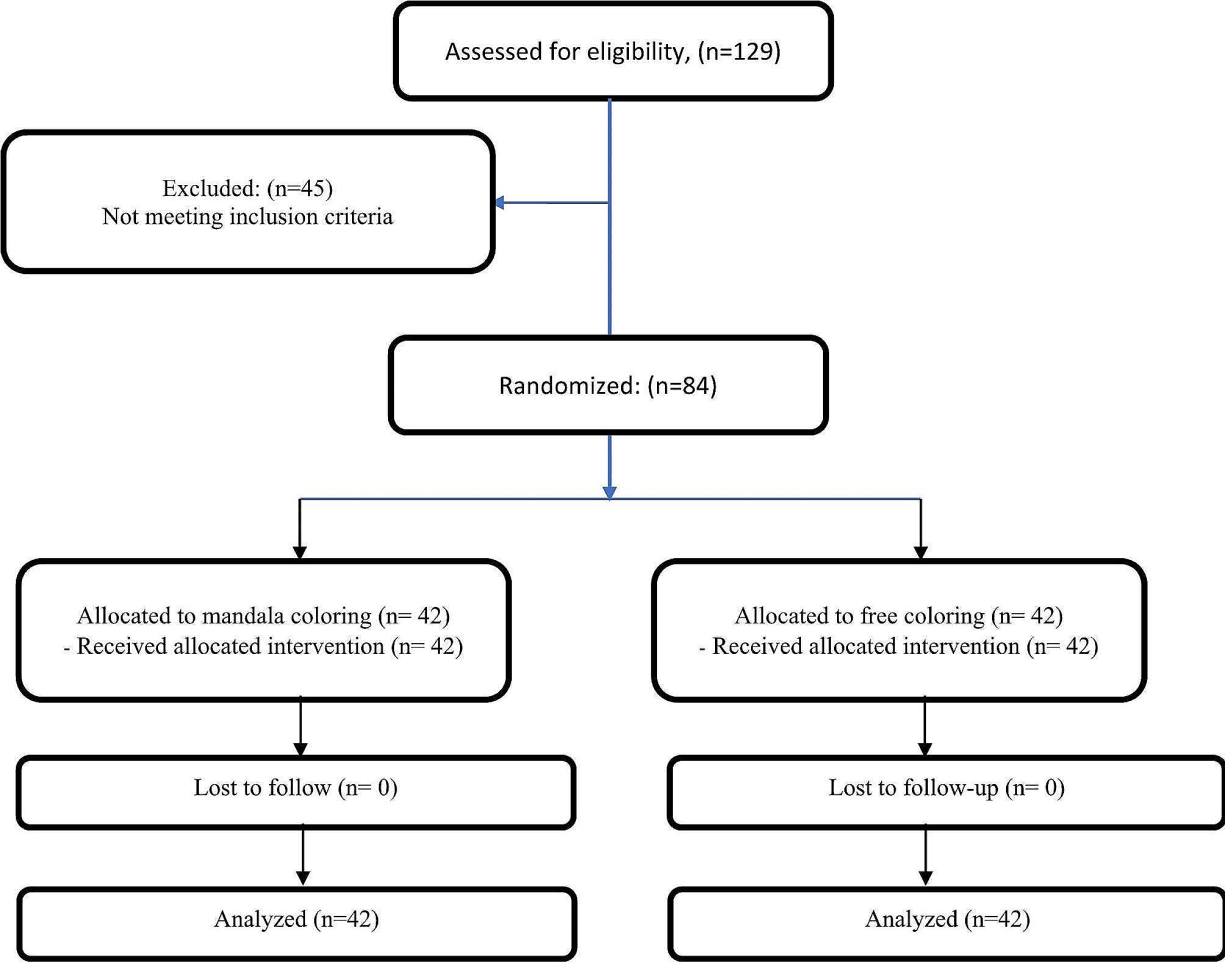
The study flow diagram

SPSS software version 16 was used for data analysis. The Kolmogorov-Smirnov test was used to examine the normal distribution of the data. Chi-square and Fisher’s exact tests were used to check the homogeneity of the two groups in terms of categorical variables. Independent sample *t*-test was used to compare the mean scores of PTSD and happiness between the two groups. Paired *t*-test was used for within-group comparison of the mean scores of happiness. The level of significance was set at *p* < 0.05. The statistical analyst was blinded to group allocation. Figure 1 shows the study process.

## Results

The mean age of veterans was 58.45 ± 3.96 (51–67) years and they had a mean duration of suffering from PTSD of 34.06 ± 2.76 [30–40] years. Most veterans had a high school diploma (52%), 78% were married, and 63% had no physical disabilities. The mean PTSD score at the beginning of the study was 65.45 ± 5.89 in the mandala coloring group and 67.47 ± 4.66 in the free coloring group (*p* = 0.085 and both groups were in an acute state of PTSD at baseline. The two groups did not differ significantly on other individual characteristics (Table 1).

**Table 1:** Individual characteristics of the patients

Variable		Mandala coloring group	Free coloring group	Test results
		Mean ± SD	Mean ± SD	
Age (year)		57.61 ± 3.56	59.28 ± 4.20	^*^ t = 1.95, df = 82, *p*-value = 0.054
Duration of suffering from PTSD (year)		34.09 ± 2.86	35.11 ± 2.58	^*^ t = 1.71, df = 82, *p*-value = 0.090
**Variable**		**f (%)**	**f (%)**	**Test results**
Education level	Low literate	17 (40.5)	14 (33.3)	^†^Value = 1.854 *p*-value = 0.808
	High school diploma	20 (47.6)	24 (57.1)	
	Academic degrees	5 (11.9)	4 (9.5)	
Marital status	Married	35 (83.3)	31 (73.8)	^†^Value = 1.131 *p*-value = 0.426
	Single	7 (16.7)	11 (26.2)	
Number of children	0	13 (31.0)	14 (33.3)	^†^Value = 3.716 *p*-value = 0.473
	1–2	13 (31)	9 (21.5)	
	3 and more	16 (38.1)	19 (45.3)	
Physical disability	No	29 (69.0)	24 (57.1)	^†^Value = 1.278 *p*-value = 0.366
	Yes	13 (31.0)	18 (42.9)	

SD: Standard deviation, f: frequency, df: Degree of freedom, PTSD: Post traumatic stress disorder

^*^Independent sample *t-*test, ^**†**^Fisher’s exact test

The independent sample *t*-test before the intervention did not show a significant difference in the happiness scores of the mandala coloring group and the free coloring group (16.85 ± 8.92 vs. 18.50 ± 7.96, *p* = 0.376). However, at the end of the study, the mean happiness score was significantly higher in the mandala coloring group than in the free coloring group (43.45 ± 9.25 vs. 39.61 ± 8.11, *p* < 0.001) (Table 2). Based on Cohen’s method, the overall effect size of mandala coloring on the veterans’ happiness was 0.44.

**Table 2:** Comparison of veterans’ happiness scores in mandala and free coloring groups

Group	Pre - test	post - test	Paired t-test Value, df, *p*-value
	Mean ± SD	Mean ± SD	
Mandala coloring	16.85 ± 8.92	43.45 ± 9.25	t = 24.55 df = 82 *p* < 0.001
Free coloring	18.50 ± 7.96	39.61 ± 8.11	t = 22.80 df = 82 *p* < 0.001
Independent sample t-test Value, df, *p*-value	t = 0.89 df = 82 *p* = 0.376	t = 2.01 df = 82 *p* = 0.047	

SD: Standard deviation, df: Degree of freedom

At baseline most veterans in the mandala coloring group (*n* = 28) and the free coloring group (*n* = 25) had low happiness. However, after the intervention most veterans in the mandala coloring group (*n* = 20) promoted to high happiness level and most veterans in the free coloring group (*n* = 23) promoted to moderate happiness level (Table 3).

**Table 3:** Comparison of happiness levels in two groups

Level of Happiness	Pre - test		Post - test	
	Mandala coloring	Free coloring	Mandala coloring	Free coloring
	f (%)	f (%)	f (%)	f (%)
Low	28 (66.66)	25 (59.52)	4 (9.52)	8 (19.05)
Moderate	11 (26.19)	15 (35.72)	18 (42.86)	23 (54.76)
High	3 (7.15)	1 (2.38)	20 (47.62)	11 (26.19)
Very high	0	1 (2.38)	0	0

f: frequency

## Discussion

The findings showed the intervention and control groups were homogeneous in terms of individual characteristics and at the baseline, the mean score of happiness did not differ significantly. Both groups had a low level of happiness. However, after the coloring activity, both groups experienced an increase in happiness scores. The mandala coloring group had a significantly higher mean happiness score compared to the free coloring group. This suggests that mandala coloring was more successful in enhancing the happiness of PTSD patients than free coloring. The overall effect size of mandala coloring on the veterans’ happiness was about 0.44, which shows the moderate effectiveness of mandala coloring in increasing the happiness of veterans suffering from PTSD.

No studies have been found that specifically examine the impact of mandala coloring on the happiness of patients with PTSD. However, there have been studies conducted on the effects of mandala coloring on anxiety and depression in veterans with PTSD. For example, Rodak et al. conducted a study where they compared the effects of mandala coloring and free coloring on anxiety, stress, and working memory in United States veterans. They found that two 15-minute sessions of coloring mandala patterns were more effective in reducing veterans’ anxiety and stress scores compared to free coloring. However, there was no significant impact on working memory [35]. Another study by Campbell et al. compared the effects of eight blended sessions of art therapy and cognitive therapy with cognitive therapy alone on depression in American soldiers with PTSD. They found that both interventions were equally effective in reducing depression following combat-related PTSD [43]. Additionally, a study on soldiers with PTSD found that six sessions of painting for the visual narration of the traumatic event and its related feelings significantly reduced depression scores [45]. Another study focused on college students and reported that five 30-minute sessions of mandala drawing improved spirituality and perceived well-being scores [46]. On the other hand, Henderson et al. reported that while mandala coloring reduced the severity of PTSD symptoms in college students who had experienced traumatic events, it had no impact on anxiety, depression, physical symptoms, and the meaning of life [47]. Furthermore, a study of 120 students also compared the effects of coloring flowers and mandalas on happiness and reported that although students found mandalas more interesting, flowers were significantly more effective in inducing happiness and calmness. They concluded that the shape and composition of mandalas and coloring them are more attractive and interesting to people and cognitively attract more attention, but these factors may not necessarily induce happiness and calmness [48]. Considering the different population being studied in the current study (veterans) compared to the population studied in the mentioned studies (students) and the differences in background factors of each, conducting further studies in this area seems necessary.

However, the increase in happiness observed in our participants can be attributed to the direct and indirect impacts of engaging in mandala coloring. Some research has categorized mandala coloring as a form of positive psychology [47, 49]. By promoting physical and mental harmony [49, 50], helping individuals discover their strengths, enhancing self-expression, improving mood, increasing feelings of self-satisfaction, and alleviating mental pain and suffering, these interventions stimulate positive emotions and emotional equilibrium, resulting in a delightful experience [50], and ultimately fostering a sense of happiness [46]. It is believed that mandala coloring can act as a mediator between the negative emotions of a painter and the conscious and unconscious mind [46]. In addition, coloring a mandala provides an opportunity for an individual to externalize negative emotions and express them in a healthy way [46], which has been shown in studies to reduce cortisol levels as a known biological marker of psychological and physiological stress [51–53]. According to Liu et al., mandala coloring offers an outlet for emotions, thereby reducing negative emotions and enhancing overall well-being [46]. Gruber and Oepen also found that mandala coloring diverts attention from negative emotions and amplifies positive emotions [54]. Additionally, a study involving 997 American soldiers revealed an inverse correlation between the severity of PTSD symptoms and the level of happiness [12]. Consequently, it is reasonable to hypothesize that mandala coloring boosts feelings of happiness by diminishing the severity of PTSD symptoms. Therefore, it is recommended that healthcare organizations provide proper training for healthcare staff and create the necessary facilities to implement these therapeutic methods, which will lead to their widespread use in patient treatment.

### Limitations

This study had limitations. In the present study, the happiness scores of the free coloring group also improved significantly. This discovery supports a previous report which stated that coloring, regardless of the specific type or conditions, has the ability to reduce anxiety and stress [35]. Conversely, the increase in happiness scores observed in the control group can be attributed to the fact that patients in this group received standard treatment methods such as psychotherapy and medication therapy. Additionally, in line with the study’s policies, all patients regularly participated in weekly group therapy and mindfulness sessions, as well as stress management techniques like slow breathing and body scanning, which aim to alleviate disease symptoms. Therefore, the improvement in scores among patients in the control group was not far from what was expected. Moreover, we only focused on male veterans with PTSD, so the results cannot be generalized to all veterans and patients with PTSD. Additionally, it is worth noting that the psychologist responsible for conducting group therapy sessions and the researcher providing mandala coloring training were aware of the group allocation.

Although veterans in the control and intervention groups were housed in two separate wards, and art-making sessions were held separately for each group, data contamination and ward effects may have influenced the results.

Due to the limitation of the sample size in the current study, it is recommended that future studies add a control group that receives only psychological intervention without any coloring methods. This addition would allow for a more accurate comparison of the effects of each method. Furthermore, future studies incorporating an art therapist during free coloring and mandala coloring sessions may yield different and more precise results concerning the efficacy of these techniques for patients. We strongly recommend that in future studies, alongside other variables, the severity of PTSD symptoms in veterans before and after coloring be assessed and examine the hypothesis that “does mandala coloring lead to increased feelings of happiness by reducing the severity of PTSD symptoms?” Indeed, clarifying the answer to this question could provide a clearer understanding of the therapeutic potential of mandala coloring.

## Conclusion

In conclusion, this study demonstrated that both mandala coloring and free coloring increased happiness scores in veterans with PTSD. However, mandala coloring was found to be more effective in enhancing the happiness of veterans with PTSD compared to free coloring. Therefore, we recommend incorporating this method into standard treatments for veterans with PTSD. Given the limited research in this area, it is suggested that similar studies be conducted on larger sample sizes. Furthermore, investigating the effects of mandala coloring on preventing acute PTSD attacks in veterans would be beneficial.

## Data Availability

The current study has been performed in a hospital. Therefore, data of the current study cannot be published due to the participants confidential. Data might be available from the corresponding author upon reasonable request and confirmation of the hospital and participants.

## Abbreviations

OHS: Oxford Happiness Scale
PCL-5: Post-Traumatic Stress Disorder Checklist for DSM-5
PTSD: Post-Traumatic Stress Disorder
